# Diabetes is associated with impaired myocardial performance in patients without significant coronary artery disease

**DOI:** 10.1186/1475-2840-9-3

**Published:** 2010-01-18

**Authors:** Charlotte Andersson, Gunnar H Gislason, Peter Weeke, Søren Hoffmann, Peter R Hansen, Christian Torp-Pedersen, Peter Søgaard

**Affiliations:** 1 Department of Cardiology, Gentofte Hospital, University of Copenhagen, Denmark

## Abstract

**Background:**

Patients with diabetes mellitus (DM) have high risk of heart failure. Whether some of the risk is directly linked to metabolic derangements in the myocardium or whether the risk is primarily caused by coronary artery disease (CAD) and hypertension is incompletely understood. Echocardiographic tissue Doppler imaging was therefore performed in DM patients without significant CAD to examine whether DM per se influenced cardiac function.

**Methods:**

Patients with a left ventricular (LV) ejection fraction (EF) > 35% and without significant CAD, prior myocardial infarction, cardiac pacemaker, atrial fibrillation, or significant valve disease were identified from a tertiary invasive center register. DM patients were matched with controls on age, gender and presence of hypertension.

**Results:**

In total 31 patients with diabetes and 31 controls were included. Mean age was 58 ± 12 years, mean LVEF was 51 ± 7%, and 48% were women. No significant differences were found in LVEF, left atrial end systolic volume, or left ventricular dimensions. The global longitudinal strain was significantly reduced in patients with DM (15.9 ± 2.9 vs. 17.7 ± 2.9, p = 0.03), as were peak longitudinal systolic (S') and early diastolic (E') velocities (5.7 ± 1.1 vs. 6.4 ± 1.1 cm/s, p = 0.02 and 6.1 ± 1.7 vs. 7.7 ± 2.0 cm/s, p = 0.002). In multivariable regression analyses, DM remained significantly associated with impairments of S' and E', respectively.

**Conclusion:**

In patients without significant CAD, DM is associated with an impaired systolic longitudinal LV function and global diastolic dysfunction. These abnormalities are likely to be markers of adverse prognosis.

## Introduction

Patients with diabetes mellitus (DM) have an increased risk of developing heart failure compared to patients without DM[1,2]. Whether this increased risk is solely based on coronary artery disease (CAD) and hypertension, or whether some of the risk might be explained by a direct influence of DM on cardiac function (diabetic cardiomyopathy) is incompletely understood.

Diabetic cardiomyopathy has been defined as the presence of myocardial abnormalities in the absence of coronary artery disease, hypertension or other significant etiology[3]. Several experimental studies have identified changes consistent with diabetic cardiomyopathy, including microangiopathy, metabolic disturbances and myocardial fibrosis[4]. The evidence of a clinical impact of diabetic cardiomyopathy on myocardial function has increased in recent years, mainly because of refined echocardiographic methods such as tissue Doppler techniques. In a large population-based study, tissue Doppler imaging revealed impaired systolic and diastolic function in persons with DM[5], and several other observational studies of populations without any apparent heart diseases have found similar results[6-9]. However, to our knowledge, no previous study has specifically examined the influence of DM in patients where coronary angiography (CAG) was used to rule out the presence of significant CAD. The aim of the present study was therefore to assess myocardial function by echocardiographic tissue Doppler in a series of DM patients without significant CAD and compare them with matched controls.

## Methods

### Population

Since the middle of 2005, all echocardiography examinations performed at Gentofte University Hospital were digitally stored on a central server. All the echocardiograhic examinations were performed by dedicated physicians and specially trained nurses, and the majorities of available investigations were conducted according to a standardized protocol. The hospital also functions as a tertiary invasive center, and since 1999, data on consecutive coronary artery catheterizations were registered in a central database. The database held information on CAD risk profile, co-morbidities, and information on the angiography procedure and coronary pathology. For the present study, all patients with DM but without significant CAD (i.e., absence of > 50% diameter stenosis in any coronary vessel ≥ 1.5 mm in diameter) examined between the middle of 2005 and the middle of 2009 were identified from the catheterization register. As per the standard CAG database protocol at the institution, the CAG diagnosis for patients in the present study included patients with 'diffuse coronary artery disease, no significant stenoses' or 'no coronary artery disease', respectively. Patients with significant valve disease, cardiac pacemaker, prior myocardial infarction, atrial fibrillation, or left ventricular ejection fraction (LVEF) <35%, respectively, were excluded. Individual patient data were then linked to the echocardiographic database. Controls comprised patients without DM, subjected to the aforementioned exclusion criteria. Each case was matched with one control on age (± 3 years, highest priority), gender (second highest priority), and presence of hypertension (third highest priority), by using a computerized selection algoritm (the Greedy macro by Lori S. Parsons, accessed January 1, 2009 at http://www2.sas.com/proceedings/sugi26/p214-26.pdf).

The median time difference between the CAG investigation and the echocardiogram record was 0 days with the inter quartile range being -1 to 43 days. Totally 56% of the echocardiograms were performed prior to CAG investigation (median time 1 day prior to CAG) and 44% of the echocardiograms were performed after CAG (median time 48 days). Among those having a CAG performed prior to echocardiography, the time delay between the two investigations was generally at most three months, but a few patients (n = 6; 4 cases and 2 controls) with longer time intervals were included (range 95 to 564 days), as it was assured from the catheterization register that no upcoming ischemic events were registered in the mean time. There was no difference in investigational time discrepancy between patients with DM and controls.

### Echocardiographic analyses

Echocardiograms were obtained using GE Vivid 7 ultrasound system. All echocardiograms were analyzed by one single investigator (CA) using Echopac '08 software (GE Medical Systems, Norway).

#### Conventional measurements

Left ventricular end diastolic dimensions (interventricular septum wall thickness, end diastolic inner diameter and posterior wall thickness) were obtained from the parasternal long axis view. Biplane LVEF was estimated using Simpson's method and a LVEF ≥50% was considered to be preserved. Left atrial end systolic dimensions were estimated from the apical four - and two chamber views.

The diastolic function was classified from measurements of color M-mode and tissue Doppler, according to the criteria accepted by the Canadian Consensus on Diastolic Dysfunction[10].

#### Tissue Doppler imaging derived measurements

Mitral basal left ventricular velocities (longitudinal peak velocities) were obtained from two dimensional color-coded tissue Doppler image loops (offline measurements). The peak systolic (S'), early diastolic (E') and atrial (A') tissue velocities were measured in the basal segments, immediately apical to the mitral annulus. The presented values are the mean values from both sides of three apical views (i.e. four chamber, two chamber and apical long axis views).

#### Average global left ventricular longitudinal strain

Global left ventricular longitudinal strain was quantified using the Automated Function Imaging (AFI), which was based on two dimensional strain imaging. The software package worked by tracking speckles (acoustic markers), and by the use of frame-to-frame changes of the speckles, motion and velocity (and thereby maximal longitudinal systolic shortening fraction) was derived. The presented values are the average from all the three apical views.

#### Intra and inter observer variation

From the echocardiography database we randomly selected 25 patients for inter and intra observer variation analyses. Intra observer variation was as follow (median [5 ^th^;95 ^th ^percentiles]): LVEF 4.8% (0;24.1); S' 1.1% (0.3;9.7); E' 0.8% (0.2;5.2); A' 1.7% (0.3;19.6); AFI 2.2% (0.3;5.9). Corresponding inter observer variation was: LVEF 4.4% (0.9;57.7); S' 2.8% (0.0;14.6); E' 2.2% (0.2;17.2); A' 2.7% (0.4;19.2); AFI 4.3% (0.4;17.2). Bland-Altman plots revealed no signs of skewed distributed variances.

### Data analysis

Continuous variables were compared with t-test and discrete variables with chi square test. General linear models were used to investigate the influence of several factors on the peak tissue velocities where the model assumptions were fulfilled (linearity of continuous variables, homogeneity and normally distributed residuals and no interactions). All calculations were performed using SAS version 9.1 (SAS institute, Cary, North Carolina). The level of statistical significance was set at a p-value < 0.05. No statistical adjustment was made for the number of comparisons performed.

### Ethics

The study was approved by the Danish Data Protection Agency (No. 2007-41-1667). Retrospective register based studies do not need ethical approval in Denmark.

## Results

In total 2388 patients were identified from the coronary artery intervention database with a diagnosis of no significant coronary artery disease/diffuse coronary artery disease without significant stenoses. Of these, 333 had diabetes, no prior myocardial infarction, no pacemaker, no congenital heart disease, and no significant valve disease registered. After matching with the echocardiographic database, 89 remained. Of these, 31 had sufficient investigations for inclusion in the study. Main reasons for exclusions were: lack of correlation between CAG and echocardiogram investigations; atrial fibrillation; bundle branch block; and investigations without tissue Doppler records.

The characteristics of the 31 patients with diabetes and 31 controls included in analyses are presented in Table 1. Patients with diabetes had a higher prevalence of dyslipidaemia, a higher prevalence of hypertension (despite attempts at matching for hypertension) and a higher BMI, compared to patients without diabetes. Notably, no significant differences were found in LVEF, left atrial end systolic volume, or prevalence of diastolic dysfunction between patients with and without diabetes. Mitral E/E' ratio was significantly higher among patients with DM (9.9 ± 5.8 vs. 7.0 ± 1.6, p = 0.01) and global longitudinal strain was significantly lower in patients with DM (15.9 ± 2.9% vs. 17.7 ± 2.9%, p = 0.03), compared to controls.

**Table 1:** Population characteristics:

	Diabetes:	No diabetes:	p for difference:
	***n = 31***	***n = 31***	
Age (years)	58 (± 12)	58 (± 12)	0.9
Gender, male	52%	52%	1.0
BMI (kg/m ^2^)	29 (± 5)	25 (± 3)	**<0.001**
Current smoker	20%	32%	0.2
Previous smoker	45%	39%	0.6
Hypertension	71%	45%	**0.03**
Hypercholesterolaemia	84%	26%	**<0.0001**
Family history of ischemic heart disease	48%	61%	0.3
**Antidiabetic medications:**			
Insulin	35%	-	
Oral anti-diabetic medication	39%	-	
Diet	13%	-	
No treatment	13%	-	
**Coronary angiography:**			
Diffuse coronary disease, no significant stenoses	35%	29%	0.6
No coronary artery disease	65%	71%	0.6
**Final diagnosis based on coronary angiography:**			
Angina pectoris (I20.)	55%	42%	0.3
Investigation for suspect heart disease (Z03.5)	45%	58%	0.3
**Conventional echocardiography parameters:**			
Left ventricular ejection fraction (%)	49 (± 7)	52 (± 7)	0.1
Proportion of patients with an LVEF ≥50%	52%	55%	0.8
Left atrial end systolic volume (ml)	35 (± 14)	42 (± 20)	0.1
Mitral E/E' ratio	9.9 (± 5.8)	7.0 (± 1.6)	**0.01**
*Left ventricular dimensions:*			
Interventricular septum thickness, end diastolic (mm)	1.1 (± 0.2)	1.1 (± 0.3)	0.5
Interventricular diamenter, end diastolic (mm)	4.8 (± 0.8)	4.7 (± 0.6)	0.3
Posterior wall thickness, end diastolic (mm)	1.0 (± 0.2)	1.0 (± 0.1)	0.8
Classification of diastolic function *:			*overall trend test p = 0.2*
Normal	55%	75%	0.1
Diastolic dysfunction I	32%	16%	0.1
Diastolic dysfunction II	10%	3%	0.3
Diastolic dysfunction III	0%	0%	-
**Tissue Doppler parameters:**			
Peak longitudinal systolic velocity, S' (cm/s)	5.7 (± 1.1)	6.4 (± 1.1)	**0.02**
Peak longitudinal early diastolic velocity, E' (cm/s)	6.1 (± 1.7)	7.7 (± 2.0)	**0.002**
Peak longitudinal atrial velocity, A' (cm/s)	6.8 (± 1.9)	6.9 (± 1.7)	0.8
Average global longitudinal strain, AFI (%)	15.9 (± 2.9)	17.7 (± 2.9)	**0.03**

Continuous variables are presented as *mean (± standard deviation) *and discrete variables are presented as *percentage*. * = In concordance with[10].

### Tissue Doppler derived peak myocardial velocities and diabetes

**Figure 1:**
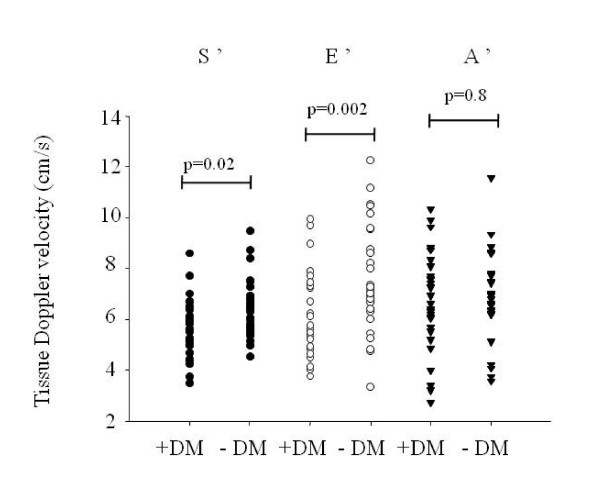
**Individual plot of mean peak systolic (S'), early diastolic (E') and atrial (A') velocities according to diabetes (DM) status**. All values are measured from the three apical views at both sides of the mitral annulus level (in total six segments).

Figure 1 illustrates the peak longitudinal systolic, early diastolic and atrial tissue velocities in patients with and without DM. Mean S' and E' were significantly lower in patients with DM, compared to controls (5.7 ± 1.1 vs. 6.4 ± 1.1 cm/s, p = 0.02 and 6.1 ± 1.7 vs. 7.7 ± 2.0 cm/s, p = 0.002).

Adjusted for multiple variables, DM remained associated with impairments in peak tissue velocities and E/E' ratio, as shown in Table 2. The presence of hypertension was not found to modify the impairments of S', E', A', or E/E' ratio associated with DM (p for interactions > 0.3 in all analyses).

**Table 2:** Estimated influence on tissue Doppler velocities:

	S' mean (cm/s)	p-value:	E' mean (cm/s)	p-value:	A' mean (cm/s)	p-value:	E/E' ratio	p-value:
**Univariable:**								
Intercept ("baseline value")	6.4 (± 0.2)	<0.0001	7.6 (± 0.3)	<0.0001	6.9 (± 0.3)	<0.0001	7.0 (± 0.8)	<0.0001
**Diabetes**	**-0.7 (± 0.3)**	**0.01**	**-1.4 (± 0.5)**	**0.004**	**0.1 (± 0.5)**	**0.8**	**2.9 (± 1.1)**	**0.02**
*R-square:*	*0.10*		*0.13*		*0.002*		*0.09*	
**Multivariable:**								
Intercept ("baseline value")	8.0 (± 1.3)	<0.0001	15.6 (± 1.6)	<0.0001	5.3 (± 2.0)	0.01	-2.9 (± 4.8)	0.6
**Diabetes**	**-0.7 (± 0.3)**	**0.04**	**-1.3 (± 0.4)**	**0.003**	**0.2 (± 0.5)**	**0.6**	**2.8 (± 1.2)**	**0.02**
Age (per 10 years increments)	-0.2 (± 0.2)	0.1	-1.2 (± 0.2)	<0.0001	0.3 (± 0.2)	0.2	1.6 (± 0.6)	0.01
Gender, male	-0.02 (± 0.3)	0.9	-0.8 (± 0.4)	0.06	0.7 (± 0.5)	0.2	0.1 (± 1.1)	0.9
Hypertension	0.02 (± 0.4)	>0.9	0.2 (± 0.5)	0.7	0.7 (± 0.6)	0.2	0.04 (± 1.3)	>0.9
CAG diagnosis: 'Diffuse coronary disease without significant stenosis', compared to 'no coronary disease'	0.04 (± 0.04)	0.9	0.09 (± 0.4)	0.8	0.6 (± 0.5)	0.2	-1.9 (± 1.1)	0.09
BMI (per 1 kg/m2 increments)	0.05 (± 0.05)	0.8	0.03 (± 0.05)	0.5	0.04 (± 0.06)	0.5	0.05 (± 0.1)	0.7
*R-Square:*	*0.15*		*0.55*		*0.17*		*0.29*	

By calculating the relative difference between patients with and without DM, the diastolic function measured by E' was shown to be the relatively most impaired of these parameters. Mean E' velocity in DM patients was only 79% of values of the control group. Correspondingly, mean S' and A' in patients with DM were 89% and 99% of the mean values of controls.

### Sensitivity analyses

Since the prevalence of hypertension was found to be higher among patients with DM, compared to controls (despite the attempted matching procedure), we performed a subgroup analysis based only on matched pairs (n = 46). These analyses showed similar results as the main study: Mean S', E' and A' were 5.9 ± 1.8 m/s, 5.4 ± 1.1 m/s and 6.3 ± 1.7 m/s in patients with DM, compared to 6.5 ± 1.2 m/s, 7.7 ± 2.0 m/s and 7.2 ± 1.8 m/s in controls, p for differences <0.001, <0.001 and 0.1, respectively.

## Discussion

The present study demonstrated that patients with DM, but without significant CAD had a reduced peak systolic and early diastolic tissue velocity as well as a reduced global longitudinal strain, compared to patients without DM. These impairments in systolic and diastolic function were not identifiable in any of the conventional echocardiographic parameters. Since all echocardiograms were obtained as a regular clinical procedure, the results of the present study are directly applicable to everyday clinical practice. To our knowledge, this is the first study investigating tissue Doppler parameters in relation to DM in patients and absence of significant CAD at CAG.

Our findings support the results from several other studies, which have found DM to be associated with impairments in systolic and diastolic function despite absence of overt heart disease[6,7,9,11,12]. Estimated by the relative difference in mean tissue Doppler longitudinal peak velocities and compared to controls, the diastolic function was shown to be more impaired than the systolic function in patients with DM. This finding is in accordance with the hypotheses of increased myocardial stiffness, increased resting myocyte tension and deposition of advanced glycated end products associated with diabetic cardiomyopathy[13]. Furthermore, myocardial steatosis, as seen in type 2 DM patients, has shown to be an independent predictor of diastolic dysfunction[14]. Because ischemia is known to initially affect the diastolic parameters[15], the observed differences could also be due to coronary microangiopathy or small vessel disease. Previous work has also demonstrated that in patients with diffuse CAD without focal stenosis at CAG, the diffuse disease process can lead to a significant continuous pressure fall along the epicardial coronary arteries, i.e., the functional equivalent of a stenosis[16]. Although there was no difference in prevalence of 'diffuse coronary artery disease, no significant stenoses' vs. 'no coronary artery disease' between patients with DM and controls, increased prevalence of atherosclerosis in patients with DM may have contributed to our findings, since these CAG diagnoses were rather subjective and ill-defined.

The presence of hypertension has previously been shown to potentiate the impairments in the diastolic function associated with DM[17]. However, the present study could not confirm these findings (no interaction was found between DM and hypertension). It is possible that our study population was too small to detect these changes, or that the hypertensive patients included in this study were pharmacologically sufficiently regulated to negate such effects.

In a clinical context, the subclinical impairments of left ventricular function in patients with DM are recognized to carry an adverse prognosis[18]. Therefore, the results from the present study further emphasize that patients with DM should continuously be regarded as a high risk group, even when CAG and conventional echocardiography investigations appear normal.

## Limitations of the study

All analyses were performed from existing echocardiogram records, which is associated with some limitations. Firstly, some patients had an echocardiography investigation carried out after CAG. Thus, there was a risk of including patients with a clinically silent but significant coronary stenosis developing in the period between CAG and echocardiogram, although no such events were recorded in the catheterization register. Secondly, patients with DM had a higher prevalence of hypertension despite our attempted matching of controls for this variable. It is not unexpected, however, that hypertension was more prevalent in patients with DM and basically it may be impossible to provide perfect matches for these patients. Thirdly, the AFI measurements were lacking in totally 12 patients (5 patients with DM and 7 controls), mainly because echocardiograms were recorded with too low frame rates, or with different heart rates in the three apical views. Therefore, AFI results were not subjected to multivariable analysis, which otherwise could have been interesting. The lack of data on HbA1c and other biochemistry parameters, diabetes duration and detailed data on medical therapies was also a limitation of the study.

## Conclusions

Patients with DM and absence of significant CAD at CAG have impaired systolic longitudinal left ventricular function and a global diastolic dysfunction, which is likely to be associated with an adverse prognosis.

## Competing interests

The authors declare that they have no competing interests.

## Authors' contributions

CA collected and analyzed the data for the present study and wrote the initial draft of the manuscript. PS, GG and SH contributed in the evaluation process of the echocardiograms. PS analyzed the echocardiograms used in inter observer variation analyses. All authors contributed substantially to study design, interpretation of the data, intellectual discussion and revision of the manuscript.
